# Culturally relevant/responsive and sustaining pedagogies in science education: theoretical perspectives and curriculum implications

**DOI:** 10.1007/s11422-021-10082-4

**Published:** 2022-02-24

**Authors:** Theila Smith, Lucy Avraamidou, Jennifer D. Adams

**Affiliations:** 1 Institute for Science Education and Communication, Faculty of Science and Engineering, University of Groningen, Room 5174.0016 (Linnaeusborg), Nijenborgh 7, 9747 AG Groningen, Netherlands; 2 University of Groningen, Groningen, Netherlands; 3 University of Calgary, Calgary, Canada

**Keywords:** Cultural adaptive pedagogies, Dutch Caribbean migrants, Community-based program, STEAM

## Abstract

The main focus of this paper is to put forward an argument about the value of asset-based, culturally relevant/responsive and sustaining pedagogies in science education, especially in former colonial contexts. Countering the framing hegemony of science education through a historically Euro-dominant lens, we call for a critical analysis of the state of science education in the Netherlands by exploring, specifically, the Dutch Caribbean community which includes Suriname and the former Dutch Antilles and Aruba and the need to respond with a more culturally relevant and sustaining pedagogical stance. In doing so, we provide a concrete example of a CR-SP-focused community-based STEAM program for young children and their parents in the north of the Netherlands. We hope that this paper will provide the foundation to springboard conversations among educators and researchers with an interest in designing, enacting and researching CR-SP-informed programs and curricula geared for historically marginalized families and children.

As one decade faded and we welcomed the dawn of another, we are still witnessing the disenfranchisement of groups of people based on *who they are*, *where they live* and *what they believe*. We witness genocide in another name and dog whistle politics that continue to frame and reframe who is considered “the Other” in society, especially in postcolonial and settler colonial contexts. For example, in the USA, the UK and Brazil, contemporary political trends have reenergized the racialized discourses that contribute to ongoing violence against Black and Brown people. In the Netherlands, which serves as the context of this paper, there exists the infamous holiday tradition of Zwarte Piet—the blackface helpers of Sinterklaas, a tradition that has embodied ridicule and degradation of Black people (Wekker, 2016). Moreover, a few European countries (i.e., France, Belgium and the Netherlands) have implemented Islamophobic policies that prohibit the use of the burqa and niqab in public spaces (Guy, 2019).

Separate from Islamophobia, we witness xenophobia at the highest levels of governments. For example, in the wake of the most recent global pandemic, in the USA, during the Trump presidency, the past-president and other top-level officials in his administration referred to COVID-19, commonly known as the coronavirus, as the “Chinese virus.” When the earliest reports surfaced that the outbreak of the virus occurred at an animal market in Wuhan, China, Asian communities in the USA reported heightened bigoted attacks where the racial epithets were certainly encouraged the onslaught of assaults (Yoshiko Kandil, 2020). This racialized and bigoted language is not new to the political arena: the Reagan-era presidential campaign saw tropes like the Welfare Queen used to caricature African American women and by extent their communities as unfairly gaining from social welfare (Bennett and Walker, 2018). Xenophobia is also evident in the turn against immigrant communities with increased deportation seen in the Western countries such as the USA, Canada, Germany and the UK (Gibney, 2008). In the UK, for instance, there are recent mass deportations of Caribbean nationals. Ironically, some of the people held by the UK Home Office are of the Windrush generation from the English-speaking Caribbean who were brought back to help rebuild Britain after the second world war (Ott, 2020). What is now dubbed the “Windrush scandal” is an indelible stain in the UK's timeline of recent happenings. In like manner, the US Immigration and Customs Enforcement (ICE) raids are carried out on a sweeping scale in immigrant communities where some of the people detained were, in the first place, seeking refuge in the USA from civil unrest and natural disasters in their home countries.

As we observe decades of Islamophobic, xenophobic and racist attacks coming to the fore during spurts of human turmoil, it is evident we have not yet slain Goliath; we have not even maimed him. The legacies of the colonial projects are still steering policies in every sector of society, education notwithstanding. This is further amplified in the science classroom where science is positioned as objective, ignoring the colonial legacy that is embedded in Western Modern Science, the science that is the foundation of the school subjects (Adams et al., 2020). With science, in general, being an understanding of the natural world it is plausible that students from different cultures will have diverse understandings of how the world works. However, experiences that are not aligned with the dominant culture of the classroom are all too often dismissed. Students’ voices are delegitimized based on a false sense of who they are or what they can contribute to the science classroom. Historically, it is the voice of the marginalized groups that are often silenced (Hooks, 1994). As a matter of fact, because science is quite often presented as neutral, judgment-free and apolitical, it masks the ongoing silencing and marginalizing of non-dominant students in science classrooms. This contributes to the longstanding issues of diversity in the sciences, especially in postcolonial and settler colonial contexts. In this paper, we argue that if we are to make science-spaces that welcome and value diverse worldviews, we need to advance a critical awareness of contextual structural inequities and then explicitly design science learning to resist and counter these systemic barriers to equitable science learning. This calls for a reconceptualization of questions related to *what is science*, *whose science*, *whose knowledge* and *science for whom?* (Harding, 1991).

## The urgency of centering non-dominant voices in science education

These questions become more relevant as the world is faced with contemporary socio-scientific challenges, such as poverty, inequalities, climate crises and global pandemics. Racialized and other marginalized groups around the globe exponentially experience the negative effects of these issues. As such, it is increasingly important for critical science educators to ensure that all voices are heard and brought to the center of scientific knowledge production and decision-making. In other words, it is important to re-center the science of being human, locating scientific meaning-making and knowledge production as connected to human lived experiences (McKittrick, 2015).

Critical researchers have been using frameworks that center non-dominant voices in science education. Norma Gonzalez, Luis Moll and others (1995) forwarded the funds of knowledge to describe the range of resources that Latino/a/x have access to in their families and communities and could be used to support student success in school. Similarly, Tara Yosso (2005) proposed the community cultural wealth knowledge to counter the deficit perspectives of students from non-dominant groups while highlighting the cultural assets that they bring into the classroom. Strong et al. (2016) outlined a critical transdisciplinary heuristic to counter neoliberal approaches to science education and expand agency for learners in ways that allow them to see layers of culture, power and knowledge in their local environments. In these asset-based approaches, researchers call for learning that centers diverse experiential perspectives, values multiple ways of knowing and advances the agency of learners from non-dominant groups.

## Purpose

In this paper, we foreground the importance of articulating and enacting asset-based pedagogies (Paris, 2012) in science teaching and learning experiences for students in the context of the Netherlands. Culturally relevant/responsive and sustaining pedagogies (CR-SP) embrace a polycultural approach to science education with an emphasis on centering and supporting marginalized students’ participation in science. The Netherlands is of special interest given its colonial past and the disengagement of the educational research community with post-colonial debates. As we will argue later on, educational research has remained neutral and apolitical despite the country’s colonial past and the multicultural nature of contemporary Dutch society. Our argument is rooted within critical theory, which offers the structure to make visible and critique the systemic oppression in the institution of education that becomes a directive force in supporting some students while marginalizing others. In this paper, we outline the history of these culturally adaptive pedagogies: culturally relevant (Ladson-Billings, 1995), culturally responsive (Gay, 2002), culturally sustaining (Paris, 2012) and culturally sustaining/revitalizing (McCarty and Lee, 2014). In tracing the history of these pedagogies, we argue for the value of using these culturally adaptive approaches for the purpose of promoting goals related to equity and social justice in science education in the Netherlands, which remain unaddressed.

### The hegemony of science and science education

With a closer, albeit, brief look at the social constructs of race and gender, we can begin to see the ways in which whiteness plays a hegemonic role in science education. Frantz Fanon (1952/2008) argued, in his book *Black Skin, White Masks,* that the Black man is striving to be white because, in essence, to be a white man is considered to be a *man*, to be human. In Katherine Mckittrick’s (2015) book, Sylvia Wynter: On being Human as Praxis, an edited collection of essays thematically centered around Sylvia Wynter’s writings and essays, she discusses the author’s explication of the yardstick of *what it means to be* and *who is* human. To be Man, is hierarchical and relational where the colonized-nonwhite-Black-poor-jobless-(female-queer) underclass is condemned to a ‘naturalized’ dysselected human status” reinforcing the socio-political oppression that pathologizes whole groups of people (Mckittrick, 2015, p.7). Kaima Glover (2016), in her review of the book, extends the description of the underclassed to include “female and queer” (p.145), which we have adopted in the preceding quote in parentheses. To deem a group of people intellectually inferior and primitive gives license to kill, rape, destroy and exploit without moral conscience. If one is less than human, then following the anthropocentric way of viewing the world, one is like an animal or plant to be dominated and commodified as a product in economic pursuit—no harm committed. Wynter (2003) pointed out that the binary human/subhuman in North America is predefined and the Category Other embodies the lack of traits and qualifications to be human and North American. We extend this definition to being human and European. The classification of being human is “to be white, of EuroAmerican culture and descent, middle class college-educated and suburban (Wynter, 1994, p.43). Therefore, the woman, immigrant, Black, Brown, ethnic white, disabled, transgender, poor “jobless school drop-out/push-out are “*perceived* to be, and *therefore behaved towards"* as the categorical other subhuman (emphasis added in original work) (Wynter, 1994, p.43).

Atwater (2000) critiques that topics on gender and feminism position white girls and women at the center of those conversations. Atwater (2000) goes so far as to assert that gender is a “code word for white females in science education” (p. 386). Her refrain of Sojourner Truth’s question of *Ain’t I woman, too?* is reminiscent of the constant struggle Women of Color, specifically Black women, face in the conversation on gender in science education. This struggle is the seemingly unheeded call of marginalized women to be considered on equal standing with their white counterparts. In the preceding argument, the use of the word gender highlighted the anatomical features of sex, female or male. However, gender can be defined as a culturally constructed biological trait, behavior and expectations of what a certain culture defines as femininity or masculinity (Brotman and Moore, 2016).

In a review study, Brotman and Moore (2008) analyzed the major shifts in gender and science over a 12-year period from 1995 to 2006. The review looked at four themes in the approach to engage girls in science. In the major shifts from the 1990s, when researchers shed light on the fact that there still needed to be major work undertaken in focusing on gender in science education, it still took years later for the discourse to examine other intersections in the conversation around gender and equity. Similarly, in her book, *Teaching to Transgress*, Hooks (1994) discusses the historical, social and political context in which Black women and white women operate and how this milieu impacts the relationship and scholarship of Black women in feminists’ works. To situate the conversation, the backdrop of the socio-historical and political context in which Black and white women operated had to be deconstructed to fully grasp the critiques Black feminist had of gender studies. Similarly, with researchers in the field of gender studies, to fully deconstruct science, we have to explore the sociopolitical and historical role of practices in the name of science in communities of minoritized groups. The call for science for *all* in the science education community is well intentioned; however, it cannot be attained if the institutionalized oppressive regime of the historically positioning of marginalized groups is not explicitly acknowledged, addressed and resisted.

In light of this critical feminist deconstruction of gender, in this paper, we offer criticism to the positioning of people of color, in particular Black people, and their relationship with the culture and practice of science. The violence against Indigenous peoples and other groups at the fringes of society as well as the erasure and appropriation without credit of knowledge, culture, practices in the name of science has to be explored in an attempt to decolonize the field of science education (Smith, 1999). The practice of Western science has been used to subjugate groups of people, deeming them primitive and less than human while at the same time appropriating their knowledge and culture, especially in the field of medicine, without giving credit. Moreover, Western Science operates around a set of values—values that influence one’s decisions on what topic to study, how to study the phenomenon, what framework one chooses to guide the analysis and interpret the findings, how one chooses to package and disseminate the knowledge (Bang, et al., 2018). All these values reinforce the ethnocentric practices that “support claims of the colonizers cultural superiority” (Bang et al., 2018, p.150). Framed under a colonial guise, these standards, unintentional as it may be, still present barriers in creating equitable and socially-just curricula design and teaching practices in science education (Adams et al., 2008).

The colonial centers of Western Europe, as Quijano (2000) asserted, profited from the violent repression of the “the colonized forms of knowledge production” and this “epistemic suppression” gave rise to the colonial extraction and reproduction of domination (p. 541). The coloniality of power through knowledge is a long-standing violent reproduction in the culture of science. Historically, Black and Brown bodies have suffered in the name of scientific advancement. The mistrust among the African American and Indigenous communities, women and the poor stems from the social institutions of race, class and gender that provided the backdrop for the ethical malpractices (Wasserman et al., 2007). Examples of this suffering are evident in the Tuskegee Syphilis Study in the 1941 cases of treatment being withheld from African American men who were infected with syphilis and the African American women who were operated on without anesthesia on by the gynecologist, hailed the “father of gynecology,” J. Marion Sims (Wasserman et al., 2007). Another example is found in a staple required reading in many science classrooms in the USA: the famous story of Henrietta Lacks whose cells were being used in research without her family’s knowledge or consent (Skloot, 2011). The abuse and violence perpetrated against Black and Brown bodies were not only observed in the USA but were also seen in other Western countries as well. In Germany, for instance, mixed-race children, born out of intimate communions between French-African soldiers and German women following the First World War, were forcibly sterilized (Dabiri, 2019). These horrid examples from the not-so-distant past reveal the underbelly of *scientific advancement* that continues to have far-reaching implications in communities of color in respect to scientific research and medicine. It is for these reasons that science education *has* to be approached from a critical perspective for the purpose of enacting a more socially just lens to disrupt racialized, gendered and classed ideologies.

### Dutch background and context

Similar to other European countries, the contemporary Dutch population is becoming more diverse and multicultural compared with the past more homogenous Dutch society. The percentage of the Dutch population with a migrant background is now 24%, and it is projected to increase to 39% by the year 2060 (CBS 2019). There are four major migrant groups in the Netherlands, with a 1-in-13 ratio of Dutch residents belonging to one of these groups (CBS, 2019). However, the population of each group is under 3%. The population breakdown includes Turkish migrants being at 2.4%, Moroccans at 2.3%, Surinamese at 2% and Dutch Caribbeans (Dutch citizens from Aruba, Curacao, Bonaire, Saint Martin, Saba and Sint Eustatius) at 0.9% (CBS Jaarrapport Integratie, 2018). We pause here to explain the complexities of the status of Caribbean Islands within the Kingdom of the Netherlands: Aruba, Curacao and Saint Martin are independent countries within the Kingdom; Bonaire, Saba and Sint Eustatius are special overseas municipalities of the Netherlands.

Swells of migration, in the Netherlands, are tied to economics, the invisible colonial machinery that still keeps the former colonies tethered to the colonial power. The increased arrival of migrants from the former colonies of the Caribbean, that is, Suriname, Aruba, the former Dutch Antilles (Curacao, Bonaire, Saint Martin, Saba and Sint Eustatius) and Indonesia, occurred in the aftermath and rebuilding of World War II (Essed, 1997). Later, migration of Surinamese, Arubans and Caribbeans of the former Dutch Antilles to the Imperial Kingdom rose with the economic downturn of the 1970s and 1980s (Essed, 1997). The presence of Moroccans and Turks is dated back to the 1960s when they emigrated to the Netherlands as migrant workers (Zorlu, 2012) during the post-world war II economic and subsequently migrant boom in northwestern Europe. Similar to the immigration pull on the former colonies—a pull to rebuild the sites of colonial power—Moroccans and Turks were filling the gap in the market for unskilled labor shortages in agriculture, industry, mining and construction in the Netherlands (de Hass, 2007). The point of interest in this paper is that of migrant students, more primarily Dutch Caribbean students, and the opportunity gap (Welner and Carter, 2013) and the educational debt (Ladson-Billings, 2006, 2007, 2013) that has accrued through “social, cultural, economic and political” (Ladson-Billings, 2007, p. 8) negligence—the onus lies with the Kingdom, not the students and their families. The opportunity gap, Welner and Carter (2013) assert, refers to the foundational disparities in society, communities and schools toward certain groups that are compounded to produce major differences in educational outcomes. Similarly, the educational debt, Gloria Ladson-Billings (2006) advances, is the deficiencies in the “historical, economic, sociopolitical and moral” (p. 5) policies and decisions that have accumulated over time. Consequently, these disparities in educational outcomes and life chances lead to significant differences in socio-economic, political and social standing, and, in the case of science fields, the loss of skills, innovative ideas and creativity.

Regardless of their colonial ties and a seemingly higher likelihood of integration based on cultural similarity and access through language, Dutch Caribbean students are more likely to be tracked into the lowest secondary school track, earning a basic certificate upon graduation. This systemic iniquitous method of tracking leaves students of Dutch Caribbean and Turkish backgrounds disproportionately represented in higher numbers in the lowest of the three secondary tracks compared to their native Dutch counterparts (CBS Jaarreport Integratie, 2018).

The term “migrant background” refers to newcomers who emigrate to the Netherlands including displaced migrants seeking asylum and refuge or voluntary migrants. In this context, someone with a migrant background extends up to the fourth generation as it is based on at least one parent having been born outside the Netherlands or who has a migrant background. On the other hand, a Dutch native is considered as someone whose parents were born in the Netherlands. This term, however, excludes those with fore-parents born outside the Netherlands as in the case of the former colonies: Suriname, Aruba, Curacao, Bonaire, Saint Martin, Saba and Sint Eustatius. This refers to the division in Dutch society of native versus migrant. The labels *autochtoon* (native) and *allochtoon* (non-native) are used to delineate between native and non-native origins (Weiner, 2014). Essentially, this means that a third-generation person of Dutch Caribbean descent, from Suriname, or the former Dutch Caribbean Antilles, is considered *allochtoon* (Weiner 2014). This outsider/insider status bestowed upon peoples from previous Dutch colonies, from countries that are still within the Kingdom of the Netherlands subjugates them to the rung of perpetual *Other* in Dutch society (Wekker, 2016). Arguably, to be *Othered* means that there is a privileged insider and disadvantaged and excluded outsider (Bennett and Walker, 2018). As Philomena Essed, one of the most prominent among the few critical race scholars in the Netherlands, and Gabriele Schwab (2012) asserted that this *otherness* is linked to the positioning of students who do not fit white Western heteronormativity as outsiders; it is an attempt to reinforce white Western heteronormativity as cultural cloning. Cultural cloning is described as the process of replicating and enforcing cultural values, practices and ways of being that privileges one group while maligning the cultures of others (Essed and Schwab, 2012).

In the course of othering and cloning, students who do not fit the dominant narrative are excluded. This is a palpable experience for Dutch Caribbean students who emigrate to the Netherlands, the mother country, in order to advance their education only to be met with this insider/outsider status, as evidenced by an Antillean young man’s account of his experience in Dutch higher education:

Through uncalled-for language corrections, ‘jokes’ based on racial stereotypes and prejudices I was and still am continuously reminded that despite my efforts to fit in, my access to the Netherlands is conditional...students from the Dutch Caribbean...say goodbye to the familiar without being entirely embraced by the new receiving society, which brings about the in-between, a third space of reality, also referred to as liminal space. (Leito 2019, p. 180)

The in-between space that Karym Leito (2019) describes reinforces the cultural imaginaries (Wekker 2016), albeit, racial and colonial imaginaries, where people from the former colonies of the empire are made to feel as “eternal foreigners” (Wekker 2016, p. 10) in the Dutch empire. A paradox of sorts, to borrow from Gloria Wekker’s description of the Dutch Imperial past. A past that has been strategically erased from the everyday consciousness of Dutch citizens. The Dutch contemporary self-representation of the open, tolerant and inclusive society is in conflict with the violent, slave-holding and colonial past (Wekker 2016). However, the presence of people from the former colonies of the East and West serves as a jarring reminder of this paradox in contemporary Dutch society.

### An overview of the Dutch schooling context

**Fig. 1:**
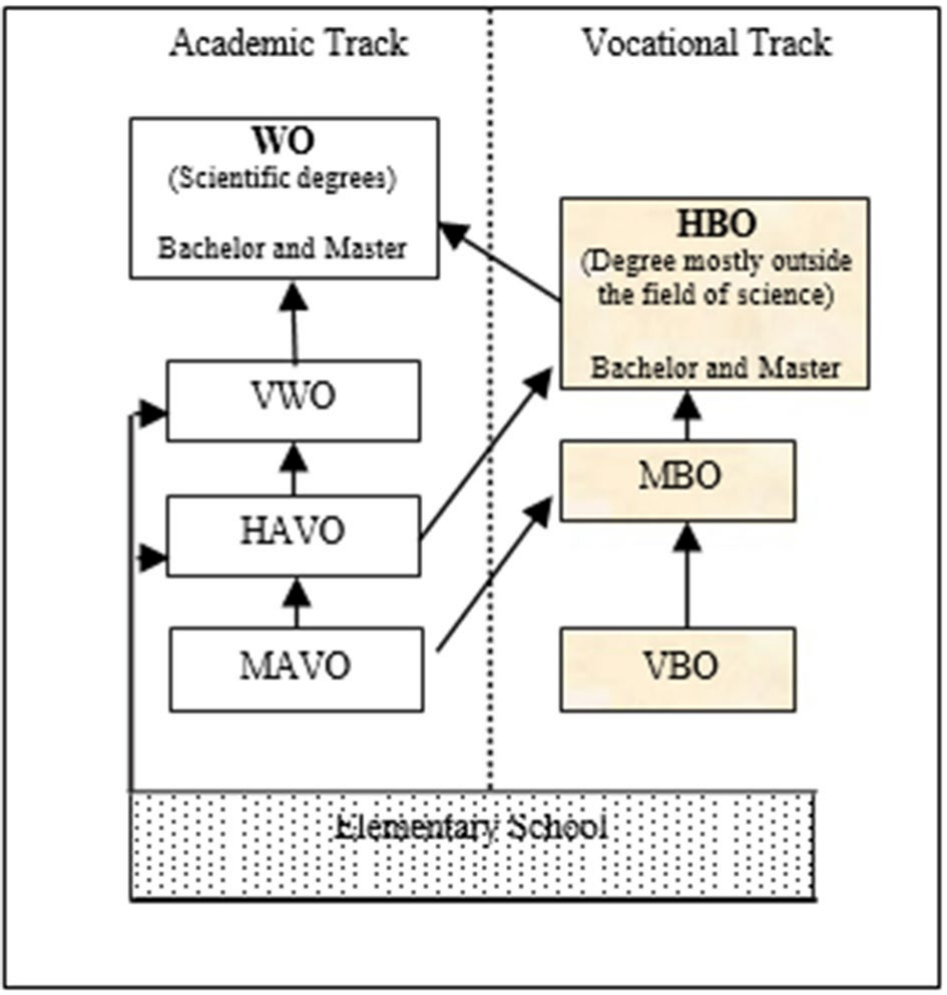
Overall structure of the Dutch educations system

Herein, laid out are descriptions of educational practices that reinforce systemic inequities that, on the surface seem supportive of students’ development, but in actuality they only serve to maintain the status quo, and consequently further marginalize and increase educational disparities between Dutch natives and, in this case, Dutch Caribbean students, in the Netherlands. On this basis, we advocate for addressing goals related to equitable and justice-oriented science education. The Dutch education system is tracked into the three disparate groups that result in different educational secondary school diplomas, which follows a binary pathway into higher education (see Fig. 1) (Zorlu, 2013). There is the pre-secondary vocational track, the lowest track, a four-year program that leads to a vocational certificate, compared to the general secondary education five-year track that leads to a professional university degree at a university or an institution of applied sciences and the third and highest track, a six-year pre-university program that affords students entrance into one of the 13 Dutch research universities (Vedder, 2006).

This figure was borrowed and adapted from Asan Zorlu’s article on the Ethnic Disparities in Higher Education (2013, p. 3).

The system of *tracking* limits students’ access to science-oriented professions. Fewer students of migrant background enter the highest track in secondary school that leads to admittance into higher degree-granting institutions to earn a professional diploma or research university. In achieving higher education degrees, students from Caribbean backgrounds, for example, tend to choose among the disciplines in law, humanities and social sciences (CBS Annual Report Integration 2018). This suggests that the likelihood of students from the Caribbean backgrounds of choosing and entering science-related fields is lower. Therefore, one could surmise that Caribbean Dutch students are underrepresented in science courses and, ultimately, science careers. Here, lack of interest on the part of the student and low social and educational capital family background (Zorlu, 2013) might explain this phenomenon. However, a counterargument is that students of migrant backgrounds including Dutch Caribbean students are granted fewer opportunities to engage with and access schools that puts them on a path to scientific careers. Additionally, fewer students from migrant backgrounds enter the highest track in secondary school that leads to admittance into higher degree-granting institutions to earn a professional diploma or research university.

Some might argue that *tracking* provides students avenues to succeed in an environment that is conducive to their needs and abilities. However, embedded within this argument, that is left unaddressed, in the case of the Netherlands, is its failure to take into account the implications of biases of low expectations, especially when these could be fraught with implicit ethnic, class and racial biases (Gilbert and Yerrick, 2001). Considering that Dutch Caribbean students, through their colonial ties, speak the language and have some intimacy with the Dutch culture should have more access to the social and cultural capital. However, the educational disparities between their Dutch native counterparts do not reflect this access. What we put forth, in this paper, is that the intricacies of being the colonized within the land of the colonizer that remains, for the most part, unacknowledged and unaddressed in education push the group to the periphery and applies to their education. Gilbert and Yerrick (2001) found, in their study with students from underrepresented groups in a lower track science class in a rural city in the USA, that the students believed that they themselves were incapable of pursuing science. Additionally, their teacher had a low perception of their students’ abilities and thought the science too rigorous for them (Gilbert and Yerrick 2001). Tracking deepens perceptions of deficit-oriented and attitudes of low expectation that supports discriminatory practices against Dutch Caribbean students and students of other migrant groups.

*Stacking* is another practice that, on the surface, is seemingly inclusive as it improves students’ chances of gaining access to higher education. On the contrary, stacking, a process that allows students to climb the certification ladder takes the student more years to obtain a degree. In this practice, a student must earn a basic qualification before moving on to the intermediate certificate to enter an applied science university, then earning a professional degree before moving onto a research institution to earn a master's degree or higher (Leito, 2019). While this shows the student’s tenacity, it takes them more years to earn degrees from higher education institutions since students first earn a vocational certificate before earning a general secondary school diploma, before they can apply to an institution of applied science (CBS Jaarrapport integratie, 2018). It must be noted that only secondary students in the pre-university track are able to gain admittance to a university; otherwise, one has to go through the stacking process outlined earlier. Comparatively, a student who enters the middle or higher track of secondary school circumvents this process of stacking by advancing straight into a professional degree-granting institution of applied sciences or a research university.

Compounded by the inherent biases in tracking and stacking practices, students from Caribbean backgrounds are less likely to study a natural science degree, as they are hampered by an educational system that limits their access. As discussed earlier, compared to their Dutch native counterparts, fewer students of migrant background gain admittance into the highest track that provides a gateway into universities where scientific research is undertaken (Netherlands Ministry of education, Culture and Science 2020). On the whole, this trend lessens the likelihood of a student from a Caribbean background pursuing a career in science. For instance, the Netherlands Centraal Bureau voor de Statistiek Statistics (CBS) 2019 annual report indicated that 21% of Turkish and 22% of Dutch Caribbean students are in the lowest track in which they earn a vocational degree upon matriculation. This percentage is countered with the paltry 8% of the Turkish and 12% of the Dutch Caribbean student population in the highest track in research universities.

The Netherlands is used in this paper as an example of a country with a colonial past, in order to make the case for the urgency and value of addressing goals related to social justice in education. This interest is rooted within the fact that the community of educational researchers, especially in the area of STEM education, have remained largely uninvolved with research that examines issues related to access, inequality, colonization and equity. As Wekker (2016) argued in her book, *White Innocence: Paradoxes of colonialism and race*, unlike North American academics, Dutch historians have persistently abstained from seeking colonial connections. Educational research in the Netherlands, we maintain, has also abstained from seeking colonial connections and examining how those shaped the curriculum. Instead, research efforts have focused solely on students’ interest, concept learning and skills development as well as the professionalization of teachers for teaching science and technology (e.g., Akkerman et al., 2019; Jansen et al., 2019; van der Wal et al., 2019; Vennix et al., 2018).

Our purpose in this paper is not to offer an exhaustive overview of the research done in the context of the Netherlands but simply to highlight the fact that some issues remain unspeakable and some questions remain unexplored. This is precisely where the contribution of this paper is found in that it calls for a thorough engagement with issues related to race and classroom-inequalities in the Netherlands, especially in the context of STEM education. Beyond this call, we also provide a concrete example of the design of a community-based STEAM program framed within culturally relevant/responsive and sustaining pedagogies (CR-SP) and targeting young children with a migrant background.

### Critical theory

Critical theory is a transdisciplinary field that has application to various fields of study, especially in the social sciences. It is also advanced as a framework in educational practice research. The goal of critical theory is to critique, theorize and undo the structure of oppression such as white supremacy that affects all areas of life, with education being of no exception (Pirbhai-Illich et al., 2017). Critical theory also underpins feminist theory, critical pedagogy and critical race theories with its offshoots of Black Crit, LatCrit, AsianCrit, QueerCrit, Tribal Crit and even critiques of whiteness, all having applications to the field of education (Dumas and Ross, 2016). Critical race theory was developed in the legal field with legal scholars such as Derrick Bell, Kimberlé Crenshaw, Richard Delgado, Mari Matsuda and Patricia Williams at the helm (Delgado and Stefancic, 2017, p. XV). Gloria Ladson-Billings and William Tate introduced critical race theory to the field of education with a strong focus on anti-Blackness in education (Dumas and Ross, 2016). Ladson-Billings, herself, coined the term culturally relevant pedagogy in response to the way African American students were being taught in integrated US schools by mostly white middle-class teachers (Ladson-Billings, 1995). Critical race theory has been used to destabilize the dominant discourse and colonizing practices that have perpetuated hegemonic “subordination of gender, class, and sexual orientation” and the persistence of racial inequities and racism (Solorzano and Yosso, 2001, p. 472). The transdisciplinary foundation of critical theory, a theory of critique, makes it applicable to the field of science education. Critical theory affords a decolonizing construct to examine the hegemony of science education.

In this paper, we propose an overarching application of critical theory to frame educational research for the purpose of decolonizing science education and cultivating a socially just society in settler colonial and former colonial contexts, such as the Netherlands. The theory includes theoretical constructs that seek to dismantle the hegemonic discourse of the dominant in science education, to challenge the status quo and to address classroom inequalities that are inextricably bound in colonialism and elitism.

## Culturally responsive education: to whose culture are we responding?

Some may say *education has always been culturally responsive.* This statement begs the question*: To whose culture are we being responsive?* (Yosso 2006). A few researchers have engaged with this question by problematizing elitist approaches to culture, education and science through the concept of “whiteness.” Bobby Habig, Preeti Gupta, Brian Levine and Jennifer Adams (2018) bring up this point when they describe how whiteness shapes diversity initiatives in informal science learning. In the US and European contexts, whiteness has been generally defined as a set of characteristics and experiences that are attached to the white race, which marks one as normal and native, while people belonging in other racial categories are perceived as “foreign” (Wynter, 2003). Whiteness has been the center of education and curriculum, and science education is no different. To examine science education, we have to examine whiteness and Eurocentrism as the dominant constructs at play given that in society, everyone is an actor enacting implicit or explicit roles based on their perceived positioning by themselves and others. Consider, for example, the role of the person receiving the action: the passive recipient of an action means there has to be a subject orchestrating that action. Thus, to be part of a group that is being marginalized then there has to be a group that is maneuvering that state of being. The marginalizer relegates the positioning of the marginalized (Cabrera et al., 2016). Critical Whiteness race scholars seek to analyze whiteness as a construct and ethnic group, going beyond the good/bad binary to unearth how whiteness as power and material property permeates all levels of society (Cabrera et al., 2016).

The word “culture” also signifies othering in education. The inclusion of cultures means there is an exclusion from the acceptable forms of knowledge. The mere sprinkling of other thoughts of being in response to inclusion suggests that there is a dominant culture that still relegates what acceptable sources of knowledge in different disciplines are.

## Historical overview of asset-based pedagogies

The call for a multicultural approach to education surfaced in the late 1960s in the USA—the period following the landmark legislation of *Brown vs. Board of Education of Topeka* 1954, the Little Rock nine (African American students integrating an all-white school), and within the era of the Civil Rights Movement. African American children were not getting a quality education even within well-resourced schools. Multicultural education emerged in the late 1960s as a countermeasure to the American school system that upheld middle-class Eurocentric values (Ladson-Billings 1994). The approach to teaching upheld the dominant culture of knowledge while making inferior the knowledge of the non-dominant. The education reform efforts championed the practice of explaining the teaching about African American culture, achievements and history. There have been countless stances on the need for education of learning and teaching to respond to the culture of the people of color in education following the 1960s into the successive decades.

Gloria Ladson-Billings used the term *culturally relevant pedagogy* (CRP) in her book, *The Dreamkeepers*. At its core is the concept of teaching and practice that honor and center African American students’ culture, values and norms in the classroom or school, especially students living in poor communities crippled by federal, state and local instituted disenfranchisement. In the USA, *culturally relevant teaching* emerged as a pedagogical stance to teaching African American students and other minoritized groups (Native peoples, Asian, Latinx, African diaspora, Brown) in the early 1990s. It has since been adopted and used by researchers in other contexts as well. Gloria Ladson-Billings’ vision of culturally relevant pedagogy is one that incorporates the family’s and community’s presence into the curriculum and school building; incites love of oneself, family and community; equip students to challenge the ideology that minoritized their culture, family, community and ways of being; and empower children to strive for excellence in every area of their lives. Culturally relevant pedagogy also extends to families—to empower families to demand the best for their children from the school board, trustees, local and state government. The major tenets of culturally relevant pedagogy/teaching (Ladson-Billings, 1995, 2009) are the following: academic success learning; sociopolitical consciousness; and cultural competence.

The definition of culture used in culturally relevant pedagogy formulated by anthropologists and sociolinguists is “the complex of explicit and tacit factors-knowledge, customs, arts, aesthetics, beliefs, language, symbols, and so on—that members of a community share and are made and remade with each generation” (Cazden and Leggett, 1976, p. 4). Rooted in Afrocentric Feminist theory, Critical Race Theory and Critical Race Feminism, CRP deviates from positioning African American students and other students of color as “deficient.” What Ladson-Billings (1995) terms succeed in doing is to address deficient pathology of assimilationist teaching, which views the education of “poor African Americans” as a means of accessing the dominant culture: white middle-class cultural norms. A culture that is seen as the truth, the one to aspire, and rendering all other cultures inferior.

## Culturally responsive teaching

Geneva Gay (2002) uses culturally responsive teaching to help teachers and teacher preparation programs “respond” to the demands of multicultural education. To create a classroom atmosphere that accommodates culturally responsive teaching, teachers need to prepare curriculum using their own cultural experiences first as a scaffold and, then, actively seek to widen their knowledge of their students’ ethnic and cultural diversity (Geneva 2002). With this broadened knowledge base, teachers can design curriculum and procedures that are culturally responsive (Gay, 2002). Geneva Gay (2013) contends that the word *culture* in culturally responsive teaching is synonymous to “values, attitudes, and beliefs; customs and traditions; heritages and contributions; and, experiences and perspectives” (p. 52) of ethnically, racially and culturally diverse students. There are four components of culturally responsive teaching that the educational researcher advocates. The first component is that teachers should adjust their attitudes and beliefs about students and communities of color. Teachers should actively work to replace pathological and deficient views of students and their communities with positive ones. Secondly, teachers should be prepared to know that there are oppositions and resistance to culturally responsive teaching. Knowing these beforehand, teachers are prepared and are able to recognize avoid or confront these differing views without distracting the work of offering culturally responsive curriculum and instruction. To this end, the third component, culturally responsive teaching should center the importance of cultural diversity in education. Finally, teachers’ curricula should reflect the local and context settings of the students (Gay, 2013).

The terms *culturally responsive* and *culturally relevant* have been used in the education literature since the 1970s in American schools and since Courtney Cazden and Ellen Leggett (1976) response to the US Supreme court’s decision in the 1974 case of *Lau v. Nichols*. The court ruled that it was unlawful under the Civil Rights Act of 1964 for San Francisco schools to deny support for Chinese American students whose first language was not English in the form of bilingual education schools. Cazden and Leggett (1976) made a case for providing and equipping teachers to address bilingual and bicultural education in schools. The terms *culturally responsive* and *relevant*, in this case, were conjoined to mean the teachers’ efforts in incorporating cultural practices of the non-dominant group in the lesson. As Courtney Cazden and Ellen Leggett (1981) argued: “Culturally responsive education rests on the fundamental nature of culture and the nature of intelligence” (p. 32). Hence, in order to respond to the “invisible” culture, schools should pay attention to the interactional styles of students and incorporate members of the community in the day-to-day operation of the school and classroom structure. However, in present society, this recommendation reinforces stereotypes as the low skill jobs in schools and classrooms are mostly occupied by individuals who do not identify with the dominant culture. There have been other pedagogical approaches that have sought to prescribe the use of the culture of the other in classroom teaching and learning practices: “culturally appropriate” (Au, 1980); culturally congruent (Mohatt and Erikson, 1981); culturally compatible (Erikson and Mohatt, 1982)” (as cited in Ladson-Billing, 1995, p. 467).

## Culturally sustaining pedagogy

Paris and Alim (2014) saw a multilinguistic practice or as Alim writes, “multilingual fluidity,” emerging from their research with students at a California high school. Students used a multitude of other languages than their own to communicate with each other. This is an example of multilingualism influenced by Hip-Hop culture and African American Language, without forsaking their cultures and languages (Paris and Alim 2014). Paris and Alim (2014) posit that educators must be explicit in their stance to sustain “languages and cultures in [educational] pedagogies in both the traditional and evolving ways they are lived and used by young people” (p. 91). CSP adds to students’ (existing) body of knowledge not to try to take away from and demean their ways of being that they encounter and enact with their families and friends and in their homes and communities. McCarty and Lee (2014) extended the work of CSP to incorporate a revitalization of the language and culture, that is, culturally sustaining/revitalizing pedagogy, a reclamation of the language when working with Indigenous youth. As they argued, in order to gain Indigenous education sovereignty, there has to be a concerted effort to reclaim the language and culture that have been stripped away from the people in the “ongoing legacies of colonization, ethnocide, and linguicide” (p.105).

One approach to applying the synergistic effect of culturally responsive–sustaining pedagogies is to create a space for the co-construction of knowledge where students’ choices and direction are central. How can this be achieved? A review of related literature shows that one such approach is through projects that allow students to choose what is contextually and culturally relevant to their local settings but not limited to the borders of their communities, town or country. In the next section, we offer a description of a community-based program in the Netherlands that is designed upon the tenets of CR-SP for the purpose of supporting young children’s development of a sense of agency.

## Curriculum implications: ROOTS: “Ik ben science!”

ROOTS: “Ik ben *Science*!” (I am science) is a research-based, STEAM enrichment, community-based program that draws on disciplines in science, technology, the arts, the environment, engineering and mathematics. ROOTS is used to refer to the call for environmental sustainability beyond geographic boundaries. “I am Science” is used to refer to students’ self-identification with science. In each session, the students are engaged in science investigations rooted within authentic problems situated in their homes and the local community, for example, testing air and water quality.

There are 25 families enrolled in the program with children ranging from 6 to 12 years old. The program is open to all children; however, the research associated with it is carried out with Dutch AfroCaribbean children. Families chose to attend on their own accord and not based on teacher or school recommendation, which we are hoping that is has eliminated the risk of further tracking of bias of who belongs in science and who does not (Avraamidou & Schwartz, 2021). The composition of families who attend cut across the socioeconomic ladder, migrant and cultural: from a single-parent student, poet and yoga teacher to a clinician who owns her own clinic. As part of ROOTS, the families get together every Saturday morning at the community center. Between the children, parents, volunteers and instructors, there are 35 people in a space which doubles as a meeting room for the staff and classroom for workshops and programs including ours. The facilitators, mostly volunteers, are a diverse group of undergraduate and graduate students as well as scientists from the alpha and beta sciences: physics, geosciences, science communication, physiology, pharmacology and psychology. Although the program is exclusively bilingual—Dutch and English—it is a multilingual reservoir of languages spoken in the group: Papiamento, Spanish, Hindi, Greek, Frisian, Afrikaanse, Zulu, siSwati, Tswana, French and German, just to name a few. The multiple languages spoken as a collective are a testament of the ethnic and cultural diversity within the group.

The site for the program is at a youth community center that serves the community in a neighborhood situated in the northern province of the Netherlands. In comparison with other districts in the urban center of the province, it is regarded as a working class and a more multicultural neighborhood in a municipality that is considered one of the poorer cities in a province that is deemed among the poorest in the country (van der Kaaden, 2017). The center provides social programs and activities for its mostly working-class clientele, one of which includes a free breakfast program for the children who attend activities at the center. The breakfast, a testament of the economic challenges some families face, is offered as some are not able to afford this for their children (Coordinator of the center, personal communication, January 28, 2020). The families who attend ROOTS come from the district as well the neighboring districts.

While a detailed description of the research component of the program is beyond the scope of this paper, in what follows we briefly discuss the research goals in order to provide a basis for understanding the rationale for the curriculum design which follows. The research component of the program is associated with the goal of employing the culturally adaptive pedagogies of responsive-sustaining pedagogies in science education, as means for supporting young children develop disruptive science identities and consequently addressing the glaring inequalities in science education in the Netherlands. Offering this program in primary education is most crucial because it is at the end of primary education that students are funneled into the three educational tracks in secondary education. As a matter of fact, there is substantial research that indicates early intervention science education in the middle and primary grades that provide students with science experiences is crucial in sustaining students’ science identification and aspirations (Kim, 2018). This evidence offers justification for the target age group of children who participate in the program. The choice of an after-school community program resonates within theories of learning and empirical evidence pointing to the fact that such spaces, unlike classrooms, provide motivating structures which have the potential to disrupt power dynamics and hierarchical relationships usually produced between students from the dominant and minoritized groups (Avraamidou and Roth, 2016).

## The goal of the program

The goal of the program is to offer a space for children and their families to come together to interrogate socio-scientific issues in their communities that they can respond with a call to action. In doing so, the program aims to create and as well as invite experiences of the families that attend, the scientists who come in to share their life histories and the volunteers who share their expertise each week.

The curriculum was designed with the input from a member of the Dutch Caribbean community who was also involved in helping to recruit families and volunteered in the sessions on Saturday mornings. The community member, Ma Troela (pseudonym) is of an older generation who has done extensive local advocacy work on behalf of the community and who had also previously ran an afterschool program at another community center in the neighborhood. The first author, who shared a similar transnational identity of being born and raised in the Caribbean, then emigrating and living in the USA and the Netherlands, met with her on a biweekly basis for five months the year prior to the start of the program. They continued over emails and text messages.

The initial meetings served as brainstorming sessions about the design of the curriculum. For example, we brainstormed lessons that included the performing and visual arts, storytelling and environmentally conscious: what we saw as culturally affirming ways of the Caribbean identity. In addition to curricular work, we would discuss political, economic and social challenges that the Caribbean community is facing in the north and the present efforts to maintain connection with cultural aspects that are distinctly Caribbean, for example, hosting events at various community centers and the junior carnival, that used to be hosted in the neighboring hood “back in the day.”

Another point of input for the curricular design was assessment interviews with parents; Ma Troela was also instrumental in helping to recruit parents for the interview. A 30-min assessment interview administered to AfroCaribbean parents six months prior to starting the program. These brief interviews were conducted to gauge the parents and the likelihood of their children’s’ interest in attending. In addition to their interests, we also sought to find out they would like to see in such a program. Subsequently, following the first five weeks of the program, we did another brief interview, an informal assessment, with the families in the program including the children to assess how they found the program and what they would like to see in activities moving forward.

The program builds on the community with parents, instructors, volunteers gathering each week to embark on an activity together. Everyone was engaged—evidenced by their fervor in completing tasks, asking and answering questions, following instructions, video-making and photographs documenting how they see science in their daily lives. Additionally, they are learning together as they build, design, and problem-solve ways to improve or test their designs.

The community goes beyond the curriculum in an act of coming together to reflect the happenings of each week and what the children, instructors and volunteers are anticipating for the weekend. This is achieved through the simple act of gathering together in a circle with the purpose in mind of cultivating a sense of trust and belonging. We want to encourage this sense of belonging, especially, to promote a collective consciousness that we are here to learn about and from each other. This is especially important in science engagement framed in CR-SP. To do that, we begin each session with a circle to reflect on our week and what we are looking forward to for the coming week. The circle process is borrowed from Kay Pranis’ (2004) restorative practices that create an environment that encourages care for each other as we pursue knowledge. Restorative practices within the restorative justice framework promote engagement and belonging in the community. The use of the circle process underpins the foundation of CR-SP: to weave together a cultural mosaic of collective belonging for all the participants in the program.

We argue for the involvement of family members as key players in supporting children in authoring their science identity. The family unit is crucial in communities that have been minoritized as extended and fictive kinships become a source of support (Alexakos, 2011). In the Black community especially, this hearkens back to traditions that were born out of a survival mechanism during enslavement but also ties to the Indigenous communities in their African homeland where the village is a central focus in helping to raise the child. This is also salient in other Indigenous peoples’ communities where the concept of the family extends beyond the immediate generations in the household like it is in Westernized society (Chatters et al., 1994).

Culturally adaptive pedagogies are incorporated into the design of the program’s curriculum. For example, one of the units focuses on a lesson on earthquakes. Though on its own a unit on earthquakes might not seem aligned with the culturally adaptive pedagogies of CR-SP; it is however, contextually relevant to the children’s and their families lives. One of the tenets of CRP along with high academic success and cultural competence is socio-political consciousness: This lesson engages children with questions about the socio-environmental impact of natural gas extraction on their and neighboring communities. Children being awakened in the middle of the night or fear of their homes being destroyed and families relocating due to earthquake tremors from natural gas extraction—an exploitation that does not directly economically benefit the northern residents—is a socio-political issue and is part of the geopolitical landscape shaping these children's lives. Culturally adaptive pedagogies not only connect to children’s experiences but also connect these lived realities to the broader contexts (Patchen and Cox-Petersen 2007).

The following excerpt is from *Een veilig huis, een veilig thuis* (2019) a report on the impact of the gas extraction on the children living in the province—the same area where the families attending program live. In the excerpt, a 12-year-old child shares with the interviewer their lack of proper rest from fear of their house collapsing while they are in a deep sleep:

Wat, ik heb zelf altijd, uhm, heb ik steeds minder diepe slaap. Ik sliep eerst altijd heel diep en daar was ik bang voor. En ik heb steeds een minder diepe slaap en uhm, dat zeg, dan denk ik van, als het trilt dan voel ik het wel. Ja dan kan ik gewoon iedereen wakker maken. Dan kunnen we naar buiten of zo. Dan kunnen we weg.

What, I always have, uhm, I have less and less deep sleep. I always slept at first very deep and I was afraid of that. And I keep having a less deep sleep and uhm, say that, then I think, if it vibrates then I can feel it. Yes, then I can just wake everyone up. Then we can go outside or something. Then we can go.

(Zijlstra Zijlstra et al., 2019, p. 5)

The occurrence of earthquakes in the northern provinces and areas close to where the program is situated, in a country which geographically should not experience this phenomenon, are quite prevalent and has far-reaching social and ecological impact on the population (Melanie et al., 2018). The continuous exploitation of this natural resource leaves the already vulnerable residents in a pernicious cycle as the earthquakes damage infrastructure including private homes, leaving frustrated homeowners to deal with damages and the cumbersome reporting system (Postmes et al. 2020).

The five-part-lesson unit features the questions of what, why and how: (a) What is happening? (b)Why is it happening? (c) Who is benefitting from this venture? (d) What are the social and environmental implications of such acts? (e) Can it be prevented? and (f) What actions do we need to take to alleviate the situation? The unit has been developed by a diverse group of women with different interests and experiences. The interdisciplinary team includes a Dutch social scientist, a South African Environmental and Infrastructure planner, an Afro-Caribbean educator and biracial Bolivian and Dutch of Indigenous heritage documentarian/activist whose work centers Indigenous communities and collective activism around environmental injustices. In both the first and second lessons, the children explored what earthquakes are, what causes them and why we experience earthquakes in the north; lastly made a model of sandstone geology layers of the decompressed and gas extraction. In the third lesson, they discussed ways they have experienced earthquakes and watched a brief documentary (CODE ROOD 2018) directed by the Dutch Bolivian documentarian on the history of gas extraction and earthquakes in the area. They also read *Een veilig huis, een veilig thuis?* (2019), a booklet—as part of a larger report—intended for a young audience, compiled of drawings and stories of young children and teenagers, who like them live in the north, documenting their experiences with the psychological and physical effects of the earthquakes. In the fourth lesson the children performed a song and dance on the impact of gas extraction on the community choreographed by an AfroCuban music and dance teacher. In the fifth and last lesson, the children were introduced to the work of the documentarian and activist, as well as learned how to write a script and tips on making their own videos. On their own, the children developed the script and prepared videos that served as public service announcements to build awareness about the earthquakes that are happening in their province. Examples of the final products the children made ranged from an investigative exploration, to the damage of homes in their neighborhood, a simulation of tremors caused by earthquakes, to a demonstration of safety precautions during earthquakes. Their videos were presented on the last day of the program with families and friends.

Another example of CR-SP in action, in addition to children's agency, is conducting Do-it-Yourself science investigations from Smart Kids Lab (http://smartkidslab.nl/). Some experiments were environmentally-based with an investigation of the pollution level of the water in the dams in their neighborhood, science in the kitchen, noise pollution in the neighborhood. A culturally affirming way the children were taught to deliver their experiments results is through storytelling. The children were introduced to storytelling by a Kenyan-born storyteller who uses African folktales (African Folktales Project) to tell stories about science. The storyteller narrated a story entitled “The First Rainbow.” The story was about a young girl who was curious about how rainbows are formed and went about her day in wonderment at seeing different colors of the rainbow in elements of her day-to-day activity. In the story, her aunt shared the story of the first rainbow, which is an African folktale:

There was a little boy called Toto, who brought the colors of the rainbow to the land of Tonota. For a long time, people in Tonota only knew the green of the plants and the brown of the soil. But Toto had vivid dreams of clouds in glorious colors! He told Mzee, the elder, about his dreams, and he said, ‘If you can name the colors, I can bring them to life.’ Then Mzee called all the rainmakers across the land, and they joined hands and formed a circle, waiting for Toto to name the colors. Toto began paying attention to his dreams. Then one by one, the colors appeared brightly. ‘Red, Blue, Orange, Yellow, Purple, Green, and Indigo!’ Toto named them all. The colorful clouds filled the sky and formed the shape of an arc! Everyone cheered and rejoiced. This was Tonota’s first rainbow. Then, Toto looked carefully at the arc of colors, and at the end of the rainbow, he noticed a pot. It was filled with gold! And since then, only Toto has seen the pot of gold at the end of the rainbow, said Aunty.

(W. Hoffman, personal communication, November 21, 2020).

The author then shared tips on how the children can write their own stories and best strategies on how to present them. Not only was the lesson affirming of the culture of Africans and people of African descent—AfroCaribbean, but it also affirmed the ancestral stories and featured cultural aspects of community in illustrating the foods we eat like mangoes and fried chicken, and the extended family systems characteristic of people of African descent, e.g., the Aunt was the main authority figure in the story, not the parents.

Table 1 outlines how the theme of each unit reflects the tenets of the culturally responsive-sustaining pedagogies.

## Conclusion

A range of frameworks have been utilized in the last two decades to conceptualize issues of globalization and multiculturalism: critical theory, feminist theory, post-structuralism and postcolonial theory (Zembylas and Avraamidou 2008). Postcolonialism especially has offered a useful lens and created unique opportunities for science educators to gain a richer understanding of globalization and multiculturalism by challenging reform efforts and science education pedagogies. This is precisely, as we argued in this paper, where the contribution of CR-SP can be found: it offers a constructive framework that problematizes various aspects of the current social and cultural conditions of science teaching and learning and it promises to make meaningful and transformative changes to science education that promote goals related to diversity, inclusion and social justice.

Our purpose, in this paper, was to explore conceptual links between asset-based pedagogies: culturally relevant, responsive and sustaining pedagogies. Essentially, what we aimed to do was to examine how these conceptual frameworks might be implemented in practice for the design and enactment of curriculum materials in the context of a former colony. Our purpose was not to dig deep into the country’s colonial past and how that has dominated the curriculum and educational policies. Instead, our purpose was to provide a concrete example of a specially-designed, community-based, after-school, STEAM enrichment program for ethnically-minoritized children: to offer “an image of the possible.”

As described earlier, the program incorporates culturally adaptive pedagogies for the purpose of improving learning opportunities and tools for all children to make sense of the world they live in. A crucial point to note is that enacting CR-SP into an out-of-school science engagement, as it is the case for classroom science, presents challenges for all stakeholders: curriculum designer/instructor, facilitators, tutors and families. As we trouble existing paradigm, we acknowledge that there are tensions in adopting what is CR-SP for the children and their families. In our case, even with two of the authors who identify as Anglophone AfroCaribbean, we realize that with the hybridized and diasporic identities of being Dutch AfroCaribbeans, we have to constantly mind the existing challenge of not overreaching and imposing what we think is considered relevant to the children in the program.

Another side to this discussion is associated with the challenges that science educators face when implementing CR-SP into the curricular design for out-of-school science programs. One of the main challenges is the theory-to-practice component of research focused on CR-SP ( Brown et al. 2019). What Megan Bang and Douglas Medin (2010) cautioned a decade ago still holds true today: “Developing culturally-based science curricula is far from straightforward” (p. 1015). That being so, the question raised is one of how do informal science educators move from the realm of theory to offering CR-SP-enriched curricula in their programs? The overriding truth, as they urged, is to address the “core problems of culture in science and science education and recognize the embedding of culture in everyday science practices” as the way forward. Roots: “Ik ben Science!” as a model brought to the fore considerations for other science educators to think about ways to engage families’ involvement and have a fully integrated CR-SP-enriched out-of-school STEAM program. Despite these tensions, it is of utmost importance that we are consistently moving toward incorporating culturally adaptive pedagogies into our curricular design.

Science education that champions science *for all* without examining, challenging and dismantling long-held practices of colonial projects, forever locks itself into a system of inequality. The call for curricula development and educational policies in science education to adopt more culturally adaptive models strikes at the root of the problem instead of treating the symptoms. This is precisely what ROOTS aims to do through engaging children and their parents in investigations about socio-scientific issues that are directly relevant to their everyday lives, as for example, the social impact of earthquakes. Hence, the activities serve as a means for engaging students in reasoning about data and sense-making as well as a means for including and embracing students’ diverse ways of reasoning and appropriating scientific vocabulary. At the same time, the activities invite students to bring in their histories, diversities, and stories as a means for embracing their roots, cultural values, and family capital.

Given the rapidly increasing ethnic, cultural, and linguistic diversity in the world, we urge science education researchers to consider goals related to supporting non-dominant students to engage with science in authentic and culturally relevant ways for the purpose of supporting them in not only seeing themselves as science persons but in also seeing themselves as agents of change. ROOTS provides a concrete example of a program that addresses such goals. We hope that this paper will provide the basis for conversations among educators and researchers interested in designing, enacting, and researching CR-SP-informed programs and curricula that aim to support students who have traditionally been marginalized and excluded from science in positioning themselves as insiders into the practice of science.

**Table 1:** Curriculum themes

Theme	Learning Goals	Activities	CR-SP tenets/design dimensions
Physics and engineering	Through the activities provided, the children will engage in activities around renewable energy and engineering concepts. Each activity is a design investigation where they will explore how they can change variables in the mini-project to see how they impact their measurable variable, including robotics and building a microphone with material physicists	In the north of the Netherlands the children might see different forms of renewable energy being used. For example, we construct and compare the speed of the hovercrafts on different surfaces. The students will explore the force of friction caused by different surfaces. The following week we design and build wind turbines and follow by using solar power to power their insects made from renewable materials	CR-SP includes the topics that are relevant to the children’s lives
Arts and Science	Through the incorporation of the arts, the children will explore different environmental themes using theatrical improvization and dancing. The children can also use the computer programming Scratch to create their animations	The activities incorporate performing and visual arts	CR-SP fosters a sense of belonging that includes not only the content but an interdisciplinary approach to the sciences that incorporate the traditions of a more communal society. For example, the movement and arts that are often seen as separate from the sciences are included in a science program
The Environment	six-part lessons centered around the earthquakes happening in the north as a result of gas extraction Why is it happening? Who is benefitting from this venture? What are the social and environmental implications of such acts? Can it be prevented? What actions do we need to take to alleviate the situation?	Activities about earthquakes (e.g., the role of plate boundaries, seismic waves, measurements. All about earthquakes in Groningen and in the home countries of the parent generations Social and Environmental Impact Build Awareness. Creating Public Service Announcements around the issue	Recognizing the political and environmental impact of the earthquakes happening in their province is crucial to their experiences. CR-SP as a practice encourages the inclusion of critical awareness of what is happening in the children’s environment
Personal Design Investigations	Children develop their own investigations. From these investigations, they will create presentations on their results for their families and friends	Create a space for the co-construction and co-creation of the issues students want to investigate in their neighborhood. These are contextual and relevant to their lives and they are the ones who would know what and how they want to address these issues	CR-SP emphasizes the need to have students tap into their creativity and resources to innovate ways of addressing issues that they encounter in their immediate and regional contexts
